# Transsynaptic BMP Signaling Regulates Fine-Scale Topography between Adjacent Sensory Neurons

**DOI:** 10.1523/ENEURO.0322-24.2024

**Published:** 2024-08-23

**Authors:** Takuya Kaneko, Ruonan Li, Qun He, Limin Yang, Bing Ye

**Affiliations:** ^1^Life Sciences Institute and Department of Cell and Developmental Biology, University of Michigan, Ann Arbor, Michigan 48109; ^2^School of Medicine, Dalian University, Dalian 116622, China

**Keywords:** axon, BMP signaling, *Drosophila*, sensory, topography

## Abstract

Sensory axons projecting to the central nervous system are organized into topographic maps that represent the locations of sensory stimuli. In some sensory systems, even adjacent sensory axons are arranged topographically, forming “fine-scale” topographic maps. Although several broad molecular gradients are known to instruct coarse topography, we know little about the molecular signaling that regulates fine-scale topography at the level of two adjacent axons. Here, we provide evidence that transsynaptic bone morphogenetic protein (BMP) signaling mediates local interneuronal communication to regulate fine-scale topography in the nociceptive system of *Drosophila* larvae. We first show that the topographic separation of the axon terminals of adjacent nociceptors requires their common postsynaptic target, the A08n neurons. This phenotype is recapitulated by knockdown of the BMP ligand, Decapentaplegic (Dpp), in these neurons. In addition, removing the Type 2 BMP receptors or their effector (Mad transcription factor) in single nociceptors impairs the fine-scale topography, suggesting the contribution of BMP signaling originated from A08n. This signaling is likely mediated by phospho-Mad in the presynaptic terminals of nociceptors to ensure local interneuronal communication. Finally, reducing Dpp levels in A08n reduces the nociceptor-A08n synaptic contacts. Our data support that transsynaptic BMP signaling establishes the fine-scale topography by facilitating the formation of topographically correct synapses. Local BMP signaling for synapse formation may be a developmental strategy that independently regulates neighboring axon terminals for fine-scale topography.

## Significance Statement

Sensory axons projecting to the central nervous system (CNS) are organized spatially to represent the locations of sensory stimuli. This occurs even between adjacent sensory axons. While much has been learned about the rough spatial arrangement of sensory axons in the CNS, the molecular signaling that arranges two adjacent axons remains poorly understood. The present study shows that this process is regulated by local interneuronal communication via a transsynaptic bone morphogenetic protein signaling that facilitates the synapse formation of the sensory axons that are appropriately located.

## Introduction

The nervous system responds differently to the same sensory stimulus presented at different locations across the body. This ability can be attributed to positional information about the sensory stimuli, which is encoded in topographic maps in the central nervous system (CNS; Sperry, 1943; Killackey, 1973; Knudsen and Konishi, 1978; Luo and Flanagan, 2007; Kaneko and Ye, 2015). Topographic maps in sensory systems are alignments of sensory afferents according to the positions of dendritic arbors. In the amphibian visual system, for instance, the retinotopic map in the tectum preserves the spatial arrangement of the dendrites of retinal ganglion cells (RGCs) by placing the axons of nasal RGCs posteriorly and those of temporal RGCs anteriorly (Goodman and Shatz, 1993; Ruthazer and Cline, 2004). Importantly, sensory neurons establish topographic arrangements even at the level of two adjacent neurons (Clandinin and Zipursky, 2000, 2002; Yang et al., 2014). This local topographic mapping, or “fine-scale topography,” allows the nervous system to distinguish sensory cues stimulating neighboring body locations at close proximity.

The molecular mechanism underlying fine-scale topography is poorly understood (Kaneko and Ye, 2015). Although broad gradients of cell-surface proteins have been identified as general guidance cues for coarse topography (Cheng et al., 1995; Luo and Flanagan, 2007), these global gradients are insufficient for molecularly discriminating neighboring neurons for fine-scale topography (Cline, 1991). Instead, spontaneous or sensory-input-evoked neural activity refines the initially coarse topography to establish fine-scale topography in systems such as the retinotopic map. In these systems, the presynaptic terminals of neighboring neurons intermingle early in development, resulting in transient connections that are topographically incorrect. Subsequently, neural activity in these sensory afferents eliminates the topographically incorrect synapses while strengthening the topographically correct connections (Cline et al., 2023). For example, the activity of glutamatergic synapses between RGC axons and their postsynaptic targets is essential for the synaptic rearrangement that establishes fine-scale topography in the amphibian visual system (Cline, 1991). Although these studies demonstrate that the communication between pre- and postsynaptic neurons plays a key role in the establishment of fine-scale topography, molecular mediators of the transsynaptic communication underlying fine-scale topography is poorly understood. In particular, how postsynaptic targets contribute to establishing fine-scale topography of presynaptic axons remains undefined.

A well characterized mediator of retrograde transsynaptic communication is the bone morphogenetic protein (BMP) signaling at the *Drosophila* neuromuscular junction (NMJ; Marques and Zhang, 2006). The *Drosophila* BMP ligand, Glass boat bottom (Gbb), is expressed in postsynaptic muscle cells and released into the synaptic cleft formed with presynaptic motor axons. Gbb binds to the Type 2 BMP receptor, Wishful thinking (Wit), on the surface of the presynaptic terminals of motor neurons, leading to the phosphorylation and activation of the BMP effector Mothers against Decapentaplegic (Dpp; Mad) inside the presynaptic terminals. A major role of retrograde BMP signaling is to establish mutual communication across synapses and coordinate pre- and postsynaptic development during synapse formation and growth. This effect of BMP signaling is mediated by phosphorylated Mad (pMad) in both the nucleus and presynaptic terminals (Berke et al., 2013, 2019; Sulkowski et al., 2014). The finding that BMP retrograde signaling regulates synaptic connectivity raises the possibility that this signaling mechanism contributes to establishing fine-scale topography.

**Figure 1.**
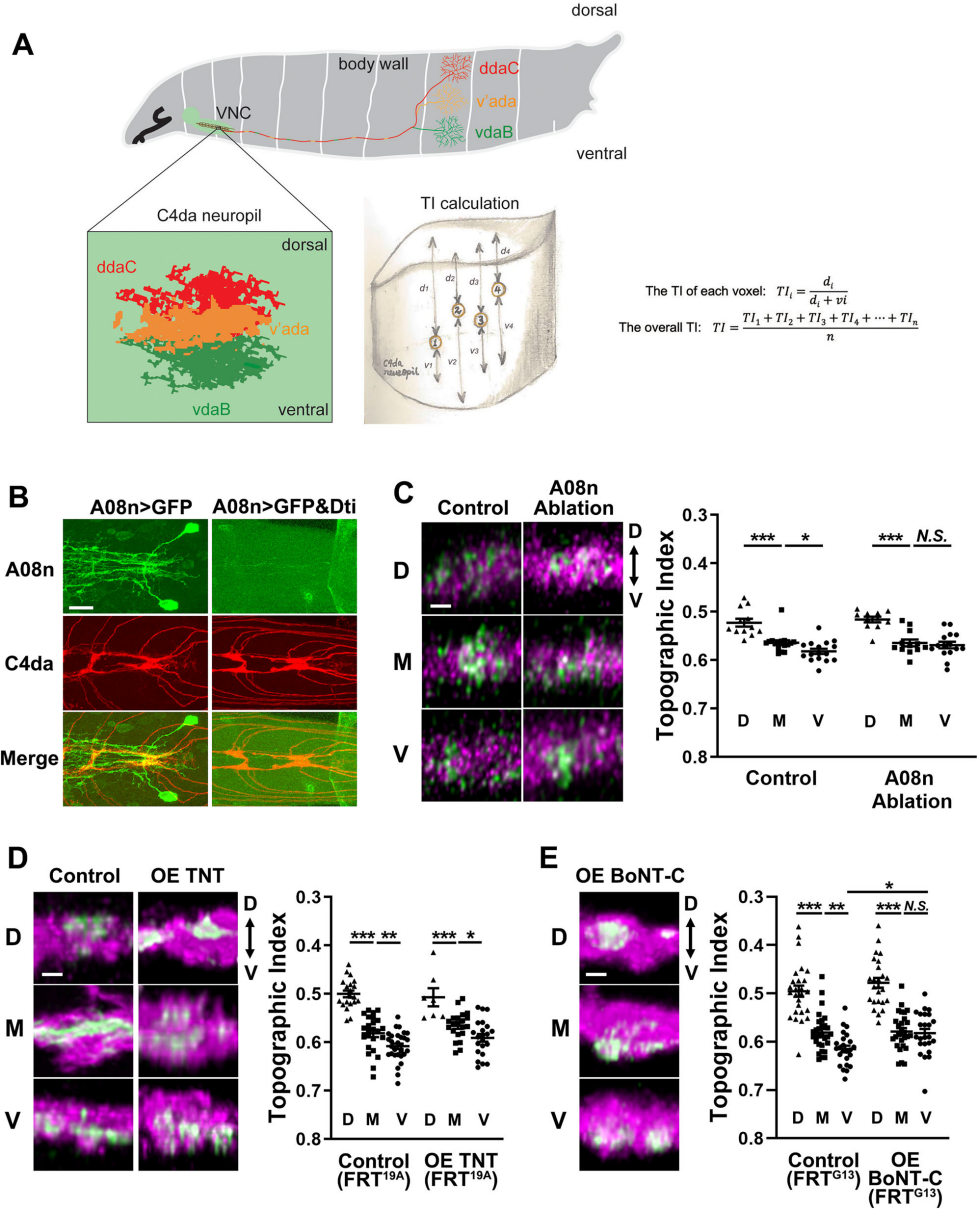
The topographic separation of M and V axon terminals requires the postsynaptic A08n neuron and spontaneous neurotransmitter release from the C4da neurons. ***A***, A schematic diagram of the activity-dependent fine-scale topographic map of C4da neurons in *Drosophila* larvae. After the axons of the three C4da neurons in each hemisegment reach the VNC, the D axon (red) separates from the M (dark yellow) and V (green) axons and projects to the dorsal portion of the C4da neuropil. The axons of M and V then separate from each other (bottom left), occupying the medial and ventral portions of the C4da neuropil, respectively. The method for calculating the TI is shown in the drawing (bottom right). Four voxels of a theoretical M neuron are shown as examples in the drawing. ***B***, Genetic ablation of A08n neurons by Dti. A08n dendrites (green) project to the C4da neuropil (red). Single C4da neurons were labeled through the flip-out technique. Dti expression by the A08n driver 82E12-GAL4 eliminates A08n neurons. Scale bars, 10 µm. ***C***, The genetic ablation of A08n neurons disrupts the topographic separation of the M and V axons but not that of the D axons. Left, Representative images of the locations of the synaptic terminals of a single GFP + C4da (green) with those of all C4da in the neuropil (magenta). Right, The TI of GFP + single C4da neurons (D, M, and V) from control animals with intact A08n neurons and A08n-ablated animals. Lower TI values represent more dorsal positioning of the axon terminals. Error bars, mean ± SEM. Throughout the paper, one-way ANOVA was used for three-group analysis with post hoc Fisher's LSD test. ***p < 0.0001; *p < 0.05; *N.S.*, *p* > 0.05. Scale bars, 2 µm. ***D***, Disruption of evoked neurotransmitter release by expressing TNT in C4da neurons does not affect the fine-scale topography. TNT was expressed in single C4da neurons with MARCM. Both groups used FRT^19A^ for MARCM. Error bars, mean ± SEM. ***p < 0.0001; **p < 0.01; *p < 0.05; *N.S.*, *p* > 0.05. Scale bars, 2 µm. ***E*,** Disruption of both evoked and spontaneous release by expressing BoNT-C disrupts topography. BoNT-C was expressed in single C4da neurons with MARCM. Both groups used FRT^G13^ for MARCM. Error bars, mean ± SEM. ***p < 0.0001; **p < 0.01; *p < 0.05; *N.S.*, *p* > 0.05. Scale bars, 2 µm. See Extended Data Figure 1-1 for more details.

In this study, we test the hypothesis that BMP signaling originated from postsynaptic neurons regulates topographic mapping at the level of two adjacent sensory neurons in the nociceptive system in *Drosophila* larvae. The larval nociceptive system detects noxious stimuli from the environment, including intense radiation, harsh mechanical stimuli, and noxious heat and chemicals (Tracey et al., 2003; Hwang et al., 2007; Xiang et al., 2010; Zhong et al., 2010; Robertson et al., 2013). These stimuli are detected by nociceptors on the larval body wall, which are called class IV dendritic arborization (C4da) neurons (Hwang et al., 2007; Zhong et al., 2010). C4da neurons elaborate their dendrites on the body wall and project their axons to the ventral nerve cord (VNC), which is equivalent to the vertebrate spinal cord. We previously showed that C4da neurons in a hemisegment of the body wall align their presynaptic terminals topographically inside the VNC (Yang et al., 2014; Fig. 1*A*). Each hemisegment is covered by dendrites of three C4da neurons, ddaC (D), v’ada (M), and vdaB (V), which innervate the dorsal, middle, and ventral portions of the hemisegment, respectively. The presynaptic terminals of the three C4da neurons are arranged in a corresponding order along the D–V axis in a compact synaptic area called “C4da neuropil.” This fine-scale topography was later confirmed by electron microscopy (EM) reconstruction of synaptic connectivity (Gerhard et al., 2017).

10.1523/ENEURO.0322-24.2024.f1-1 Figure 1-1 Genetic ablation of A08n neurons by Dti does not affect the morphology of the C4da presynaptic terminals. Single C4da neurons are labeled in green, while all C4da neurons are marked in red. Scale bars: 5  µm. Download Figure 1-1, TIF file.

The axons of the three C4da neurons in each hemisegment are fasciculated into a single nerve bundle that enters the VNC. These three axons are then sorted topographically through at least two distinct mechanisms (Yang et al., 2014; Fig. 1*A*). First, the D axon separates from the M and V axons. The D axon enters the C4da neuropil from the dorsal side while the M and V axons do from the ventral side. Second, the M and V axons separate from each other after entering the C4da neuropil. Whereas the separation of the D axon at the first step is independent of neural activity, the topographic separation of adjacent M and V neurons at the second step depends on the activity of these neurons (Yang et al., 2014). For example, when neural activity of an M neuron is inhibited, the M axon terminates at the position where the V axon in the same hemisegment is normally situated. Together with advanced genetic techniques available in *Drosophila*, this system offers a unique opportunity for identifying the molecular mechanism in the establishment of fine-scale topography.

Here we report that establishing fine-scale topography among the C4da neurons requires A08n neurons, a common postsynaptic target of D, M, and V C4da neurons (Gerhard et al., 2017). We further show that A08n neurons regulate C4da topography through BMP signaling. A08n neurons express the BMP ligand, Dpp, which is required for C4da topography. Inhibiting BMP signaling in single C4da neurons disrupts their topographic projections. Furthermore, postsynaptically originated BMP signaling is required for synapse formation between C4da and A08n neurons. Thus, transsynaptic BMP signaling regulates fine-scale topography likely by stabilizing synaptic connections between topographically correct partners.

## Materials and Methods

### Experimental model

This study used *Drosophila melanogaster* strains, not selected based on sex. The following fly lines are used:

GAL4/LexA stocks: ppk-GAL4 (Grueber et al., 2007); ppk-LexA (Gou et al., 2014), GMR38A10-LexA (Bloomington *Drosophila* Stock Center, stock number BL-54106), GMR82E12-GAL4 (BL-40153), GMR82E12-LexA (BL-54417; Vogelstein et al., 2014); Dpp-GAL4 (BL-7007); and Gbb-lexA (Kanai et al., 2018); UAS/LexAop stocks: UAS-CD4-GFP (BL-35836); UAS-GFP::lacZ-nls (BL-6452); UAS-Dti (BL-25039); UAS-TNT (BL-28838); UAS-BoNT-C (Ramesh et al., 2021); UAS-mCherry-RNAi (BL-35785); UAS-Dpp-RNAi (BL-33767 and BL33618); UAS-Gbb-RNAi (BL-34898); UAS-Tkv^ACT^ (Berke et al., 2013); UAS-Sax^ACT^ (Berke et al., 2013); UAS-Mad^1^ (Berke et al., 2013); UAS-Put (Tian and Jiang, 2014); UAS-Wit (gift from Dr. Michael O'Connor); LexAop-FRT-STOP-FRT-mCD8-GFP (BL-57588); LexAop2-CD4-tdTomato (BL-77138); LexAop-tdTomato-nls (BL-66680); and LexAop-syb::spGFP^1-10^, UAS-CD4::spGFP^11^ (BL-64315; Macpherson et al., 2015); and other stocks: hs-flp^122^ (BL-1929); ppk-CD4-tdTomato (Yang et al., 2014); FRT^82B^, *put^P^* (Tian and Jiang, 2014); FRT^2A^, *wit^A12^* (BL-5173 recombined with FRT^2A^); and FRT^40A^, Mad^1-2^ (Berke et al., 2013).

### Experimental design and statistical analysis

#### Image processing and quantification for determining the topographic index

The topographic analyses were conducted as described previously (Yang et al., 2014). Briefly, single C4da neurons [i.e., Mosaic Analysis of Repressible Cell Marker (MARCM) or FLP-out clones] were labeled by GFP with MARCM or FLP-out, while all C4da neurons are marked by tdTomato as a reference. The images were collected as three-dimensional (3-D) stacks at 0.3 μm steps using either an FV1000 confocal system (Olympus Microsystems) equipped with a 60× oil lens (Plan-Apochromat, NA, 1.4) or a SP5 confocal system (Leica Microsystems) equipped with a 63× oil lens (Plan-Apochromat, NA, 1.4). Minimum signal saturation was ensured during image acquisition. The image stacks were deconvolved with the Huygens software (Scientific Volume Imaging). The 3-D image analysis software Amira (FEI Visualization Sciences Group) was employed to align the VNC in each stack to a uniform orientation. The 3-D image stacks containing only the C4da neuropil with the single clones were cropped by ImageJ (National Institutes of Health). After the preprocessing above, the image stacks were analyzed with the software QTIP (Quantifier for Topographic Index of Presynaptic terminals) for the topographic index (TI; Zhou et al., 2015).

The relative position along the dorsal–ventral axis of a GFP + presynaptic terminal inside the tdTomato + C4da neuropil was quantified as the TI. The TI of a clone is the average of the TI for each clone voxel (TIi) calculated for all GFP + voxels inside the C4da neuropil. TIi = di / (di + vi), where di is the distance of the dorsal most GFP signal from the dorsal most tdTomato signal (i.e., the dorsal boundary of the C4da neuropil) and vi is the distance of the ventral-most GFP signal from the ventral-most tdTomato signal (i.e., the dorsal boundary of the C4da neuropil; Fig. 1*A*). GFP + clones with dorsally located presynaptic terminals have TIs closer to 0, whereas those with ventrally located terminals have TIs closer to 1. TIs were analyzed with one-way ANOVA with post hoc Fisher's LSD test.

#### Immunostaining and quantification

Third instar larvae were dissected and stained as described previously (Kim et al., 2013; Wang et al., 2013). Primary antibodies used were mouse anti-GFP (1:100; Sigma-Aldrich; RRID, AB_259941); chicken anti-GFP (1:2,500; Aves Labs; RRID, AB_2307313); rabbit anti-RFP (1:5,000; Rockland Immunochemicals; RRID, AB_2209751); and rabbit anti-pMad (1:200; Abcam anti-Smad3; Phospho S423 + S425; Chiang et al., 2017); rat anti-elav (1:500, Developmental Studies Hybridoma Bank); and rabbit anti-Dpp (1:500, gift from Dr. Takuya Akiyama). Secondary antibodies used were (1:500, Jackson ImmunoResearch Laboratories): donkey anti-mouse Alexa Fluor 488 (RRID, AB_2340846), anti-chicken Alexa Fluor 488 (RRID, AB_2340375), anti-chicken Alexa Fluor 647 (RRID, AB_2340380), anti-rabbit Rhodamine RX (RRID, AB_2340613), anti-rat Alexa Fluor 647 (RRID, AB_2340694), and anti-rabbit Alexa Fluor 647 (RRID, AB_2492288). In Figure 4*B* and Extended Data Figure 3-1*A*, pMad was costained with an antibody against the pan-neuronal nuclear protein Elav. The images were collected using Leica SP5 with a 20× oil lens, with the same acquisition setting throughout all samples. All signals at the time of acquisition are below saturation. *Z*-stack images were collected with 0.3 µm *z*-steps for over 50 slices. Elav signals were used to identify the nucleus, and a region of interest (ROI) was drawn to outline the nucleus of a C4da neuron. The intensity of pMad signals in the ROI, measured from the max-intensity projection image with ImageJ, was normalized by that of Elav signals in the same ROI. The contribution of pMad signals from nearby cells was minimum because the nearby cells did not overlap with the C4da nucleus in the *z*-stack images analyzed. One-way ANOVA was used.

#### Synaptobrevin-GRASP and quantification

Syb-GRASP was performed as described previously (Macpherson et al., 2015; Kaneko et al., 2017). Reconstituted GFP (i.e., GRASP) signals were detected by anti-mouse monoclonal GFP antibody (Sigma-Aldrich; RRID, AB_259941; 1:100). Syb::spGFP^1-10^ was preferentially detected by anti-chicken polyclonal antibody (Aves Labs; RRID, AB_2307313; 1:2,500). Syb-GRASP signals were normalized by the level of syb-spGFP^1-10^ in C4da axons. The samples of the control and two RNAi groups were processed simultaneously, including staining in the same tube to ensure identical antibody concentration. Images were collected using an FV1000 confocal system (Olympus Microsystems) equipped with a 60× oil lens (Plan-Apochromat; NA, 1.4) at 0.3 µm *z*-steps. Signal saturation was minimized during image acquisition. The same imaging settings were applied to all samples. One-way ANOVA was used for multigroup analysis with post hoc Fisher's LSD test.

## Results

### Larval nociceptors require postsynaptic A08n neurons for fine-scale topography

A08n neurons are important postsynaptic interneurons of C4da nociceptors in *Drosophila* larvae (Vogelstein et al., 2014; C. Hu et al., 2017; Kaneko et al., 2017; Y. Hu et al., 2020). They form synaptic contacts with all three C4da subtypes, D, M, and V, at the first instar larval stage (Gerhard et al., 2017). We examined the possibility that the C4da topography requires postsynaptic targets by genetically ablating A08n neurons through the expression of diphtheria toxin (Dti) under the control of the A08n GAL4 driver GMR82E12-GAL4 (Vogelstein et al., 2014; Fig. 1*B*). The genetic ablation of A08n neurons did not change the gross morphology of C4da presynaptic terminals (Extended Data Fig. 1-1). To determine the relative locations of each terminal along the D–V axis, we measured the TI of individual C4da presynaptic terminals as described previously (Yang et al., 2014; Fig. 1*A*). In brief, the TI of each voxel (TI_i_) of the axon terminal image of a single C4da was calculated by measuring its relative position between the dorsal and ventral boundaries of the C4da neuropil. The overall TI of an axon terminal is the mean of the TI_i_ of all the voxels. In control larvae with intact A08n neurons, the TI showed segregation of D, M, and V axon terminals along the D–V axis (Fig. 1*C*; *F*_(2,41)_ = 23.29; *p *< 0.0001; one-way ANOVA; M vs V, *p *= 0.0343; Fisher's LSD). In contrast, in larvae without A08n neurons, the M and V presynaptic terminals exhibited comparable topographic indices, indicating a lack of D–V separation of the M and V terminals inside C4da neuropils (Fig. 1*C*; *F*_(2,35)_ = 17.70; *p *< 0.0001; one-way ANOVA; M vs V, *p *= 0.6428; Fisher's LSD). Ablating A08n neurons did not affect the separation of D from M axons. These results suggest that the separation of adjacent M and V axons requires the interaction between C4da axon terminals and their postsynaptic A08n neurons.

Since neural activity in C4da neurons is an instructive cue for the topography (Yang et al., 2014), we hypothesized that neurotransmitter release from C4da neurons is an essential component of the C4da→A08n interaction for fine-scale topography. We first examined the requirement of evoked neurotransmitter release by expressing tetanus toxin light chain (TNT) in single C4da neurons, which blocks evoked synaptic release and is effective for blocking synaptic transmission in C4da neurons (Sweeney et al., 1995; Robertson et al., 2013). MARCM (T. Lee and Luo, 1999) was used to simultaneously label and express the TNT transgene in single C4da neurons. C4da neurons expressing TNT exhibited normal topography (Fig. 1*D*; *F*_(2,47)_ = 14.78; *p *< 0.0001; one-way ANOVA; D vs M, *p *= 0.0005; M vs V, *p *= 0.0256; Fisher's LSD), suggesting that evoked neurotransmission is dispensable. Next, we blocked both evoked and spontaneous neurotransmitter release by expressing in single C4da neurons botulinum toxin-C (BoNT-C), which targets Syntaxin (Ramesh et al., 2021). BoNT-C expression in single V neurons dorsalized the V terminal (Fig. 1*E*, the V of control vs the V of OE BoNT-C, *p *= 0.0120) and eliminated the topographic difference between the M and V axon terminals (*F*_(2,74)_ = 45.44; *p *< 0.0001; one-way ANOVA; D vs M, *p *< 0.0001; M vs V, *p *= 0.7848; Fisher's LSD). BoNT-C expression in single D neurons did not affect the D projection. These results suggest that spontaneous, but not evoked, neurotransmitter release from the nociceptors is required for establishing fine-scale topography.

### Dpp expression in postsynaptic A08n neurons is required for c4da topography

**Figure 2.**
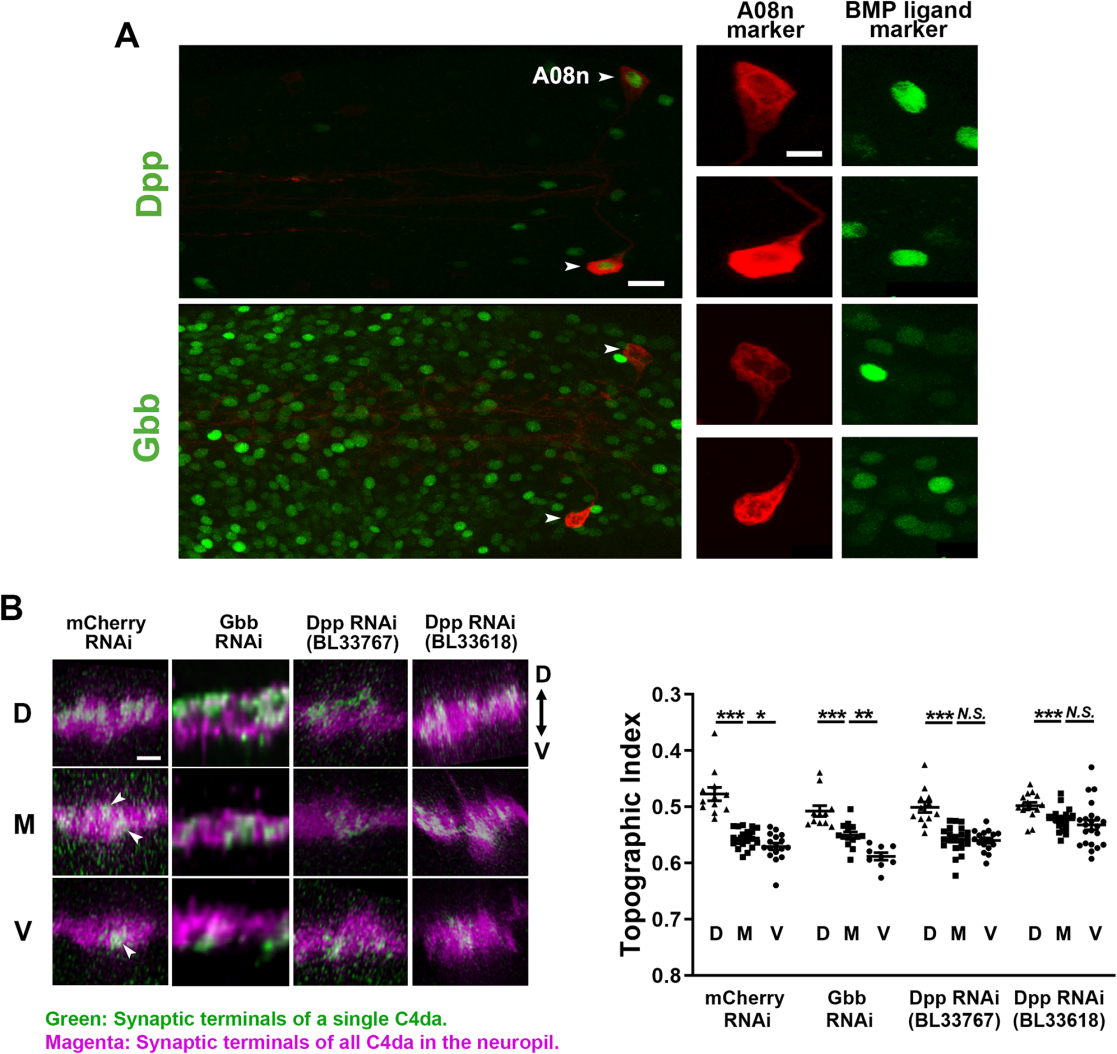
The expression of Dpp in the A08n postsynaptic neurons is required for fine-scale topographic separation of C4da axon terminals. ***A***, The expression of BMP ligands in the VNC. Dpp-GAL4 is expressed in A08n neurons (top panel), while Gbb-lexA is not (bottom panel). A08n neurons were marked by membrane fluorescent proteins (CD4-tdTomato or CD4-GFP; red) and Dpp or Gbb-positive cells were labeled by nuclear fluorescent proteins (GFP::LacZ-nls or tdTomato-nls; green). Arrowheads point at the cell bodies of A08n neurons. Right panels are single-channel images of magnified A08n cell bodies. Scale bars, 10 µm (left) and 5 µm (right). ***B***, Dpp expression in A08n neurons is required for the fine-scale separation of the M and V axons. The synaptic terminals of single C4da, labeled by flip-out, are shown in green, and those of all C4da in the neuropil are in magenta. Either mCherry-RNAi (control), Gbb-RNAi, or Dpp RNAi (BL33767 and BL33618) was expressed in A08n neurons using GMR82E12-GAL4. In the control (mCherry RNAi), the broadly distributed green voxels in the M terminals (arrowheads) had an overall TI that is close to 0.55 (i.e., around the middle between the D–V boundaries), which is different from the V neuron terminals located at the ventral-most portion of the C4da neuropil (arrowhead). Dpp RNAi eliminated the difference in the TI between the M and V axon terminals. Fisher's LSD test was used for two-group analysis. Error bars, mean ± SEM. ***p < 0.0001; **p < 0.01; *p < 0.05; *N.S.*, >0.05. Scale bar, 2 µm. See Extended Data Figure 2-1 for more details.

How might postsynaptic neurons contribute to C4da topography? We hypothesized that signaling molecules derived from the postsynaptic A08n neurons regulate C4da projections. The best characterized retrograde signaling in *Drosophila* is BMP signaling at the larval NMJ, where the BMP signaling is active in glutamatergic motor neurons in response to BMP ligands secreted from muscle cells (Marques et al., 2002, 2003; McCabe et al., 2003, 2004; Marques and Zhang, 2006). Inspired by these studies, we determined whether postsynaptic A08n neurons express any BMP ligands. The expression of two BMP ligands, Dpp and Gbb, was examined in the larval VNC by expressing a nuclear fluorescent reporter under the control of Dpp-GAL4 or Gbb-lexA driver lines (Kanai et al., 2018). Although only a small population of neurons expressed Dpp-GAL4 in the VNC, A08n neurons expressed high levels of the reporter (Fig. 2*A*). In contrast, the expression of Gbb-lexA was observed in a large population of neurons in the VNC, but not in A08n neurons. This result supports the notion that A08n neurons produce the BMP ligand Dpp.

10.1523/ENEURO.0322-24.2024.f2-1 Figure 2-1 Dpp proteins are present in A08n soma, which decrease by RNAi knockdown. (**A**) Left: Dpp immunostaining of the larval VNC. Dpp is shown in red, and A08n neurons are in green. Scale bar: 10  µm. Right: quantification of Dpp levels in A08n soma. Dpp signal intensity was normalized by GFP intensity. (**B**) 3-D reconstruction (with Amira, FEI Visualization Sciences Group) of the immunostaining data from **A**. Dpp proteins (red) can be seen with in A08n soma (green). Download Figure 2-1, TIF file.

To determine whether Dpp in A08n neurons is essential for the fine-scale C4da projections, we blocked Dpp expression specifically in A08n neurons through RNA interference (RNAi) by expressing shRNAs against Dpp (Dpp RNAi) under the control of the A08n GAL4 driver GMR82E12-GAL4 (Fig. 2*B*). While a shRNA against Gbb did not affect the topography (*F*_(2,31)_ = 26.00; *p *< 0.0001; one-way ANOVA; D vs M, *p* = 0.0002; M vs V, *p *= 0.0013; Fisher's LSD), two independent shRNAs against Dpp (BL33767 and BL33618) in A08n neurons disrupted topographic separation of the M and V presynaptic terminals (BL33767, *F*_(2,47)_ = 25.21; *p *< 0.0001; one-way ANOVA; D vs M, *p *< 0.0001; M vs V, *p *= 0.7839; Fisher's LSD; BL33618, *F*_(2,54)_ = 5.958; *p *= 0.0046; one-way ANOVA; D vs M, *p *= 0.0182; M vs V, *p *= 0.3221; Fisher's LSD), which is similar to the effect of the genetic ablation of A08n neurons.

Immunostaining with an anti-Dpp antibody showed that endogenous Dpp appeared as puncta throughout the VNC (Extended Data Fig. 2-1*A*), which is consistent with prior studies showing its staining outside of the expressing cells as the result of their release from these cells (Bauer et al., 2023). The A08n neurons did contain Dpp + puncta in their cell bodies (Extended Data Fig. 2-1*B*), which was reduced by shRNAs BL33767 (Extended Data Fig. 2-1*A*; *t *= 2.234; *p *= 0.0453; *t* test). We did not observe a significant reduction of the endogenous Dpp with the BL33618 RNAi line, which may be due to high background signals of the anti-Dpp immunostaining.

Taken together, these results suggest that A08n neurons regulate C4da topographic projections through the Dpp they release.

### Topographic separation of C4da axon terminals requires the BMP signaling in C4da neurons

Given the requirement of Dpp in postsynaptic A08n neurons for the topographic projections of C4da neurons, we predicted active BMP signaling in C4da neurons. The activation of BMP receptors upon ligand binding results in phosphorylation of the BMP effector Mad (Marques and Zhang, 2006). pMad are mostly transported to the cell nucleus to initiate transcription of target genes. Therefore, the level of BMP signaling is partly reflected by the level of nuclear pMad (Tanimoto et al., 2000). Nuclear pMad expression in the D type of C4da neurons was previously demonstrated (Follansbee et al., 2017; Honjo and Tracey, 2018). Through immunostaining with an anti-pMad antibody, we further found that pMad is expressed at similar levels in the M and V nuclei (Extended Data Fig. 3-1*A* and Fig. 4*B*), demonstrating that BMP is active in all three C4da neurons in each hemisegment.

**Figure 3.**
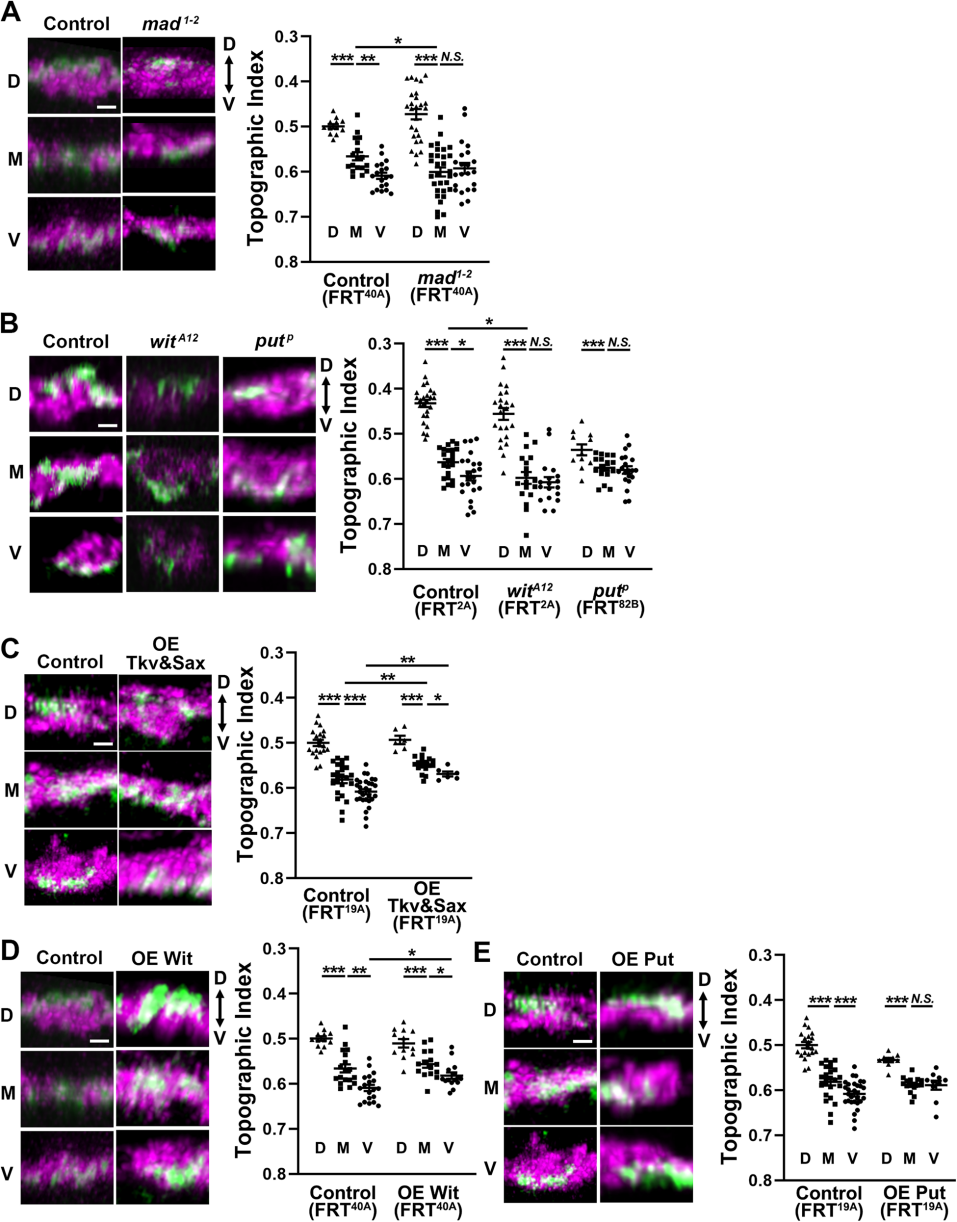
The topographic separation of C4da axon terminals requires the BMP signaling. ***A***, *Mad* deletion disrupts C4da topography. MARCM was used to manipulate single GFP + C4da neurons (green). All C4da neurons expressed tdTomato (magenta). FRT^40A^ was used for both wild-type control and *mad^1-2^* mutation in MARCM. Error bars, mean ± SEM. ***p < 0.0001; **p < 0.01; *p < 0.05; *N.S.*, *p* > 0.05. Scale bar, 2 µm. ***B***, The BMP Type 2 receptors *put* and *wit* are required for C4da topography. MARCM was used to induce loss of *put* or *wit* in single GFP + C4da neurons (green). All C4da neurons expressed tdTomato (magenta). FRT^2A^ was used for control and *wit^A12^*; FRT^82B^ was used for *put^P^* in MARCM. Error bars, mean ± SEM. ***p < 0.0001; *p < 0.05; *N.S.*, *p* > 0.05. Scale bar, 2 µm. ***C***, Hyperactive BMP signaling affects C4da topography. MARCM was used to express Tkv^ACT^&Sax^ACT^ in single C4da neurons. Single C4da neurons express GFP (green), while all C4da neurons express tdTomato (red). FRT^19A^ was used for MARCM. The control (FRT^19A^) is the same dataset used in Figure 1*D*. Error bars, mean ± SEM. ***p < 0.0001; **p < 0.01; *p < 0.05. Scale bar, 2 µm. ***D***, Wit overexpression (OE Wit) disrupts C4da topography. UAS-Wit was overexpressed in GFP + neurons by MARCM with FRT^40A^. The control (FRT^40A^) is the same dataset used in Figure 3*A*. Error bars, mean ± SEM. ***p < 0.0001; **p < 0.01; *p < 0.05. Scale bar, 2 µm. ***E***, Put overexpression (OE Put) disrupts C4da topography. UAS-Put was overexpressed in GFP + neurons by MARCM with FRT^19A^. The control (FRT^19A^) is the same dataset used in Figure 1*D*. Error bars, mean ± SEM. ***p < 0.0001; *N.S.*, *p* > 0.05. Scale bar, 2 µm. See Extended Data Figures 3-1 and 3-2 for more details.

Next, we asked whether the BMP signaling in C4da neurons is required for the C4da topographic projections. We disrupted BMP signaling by deleting *Mad* in single C4da neurons through MARCM (with the null allele *Mad^1-2^*) and then quantified the TI. We found that single M neurons homozygous for *Mad^1-2^* projected their presynaptic terminals to the ventral side of the C4da neuropil (Fig. 3*A*; M of control vs M of *Mad^1-2^*, *p *= 0.0185), eliminating the topographic difference between the M and V axon terminals (*F*_(2,38)_ = 38.05; *p *< 0.0001; one-way ANOVA; D vs M, *p *< 0.0001; M vs V, *p *= 0.9778; Fisher's LSD). D and V neurons exhibited normal projections independent of *Mad* functions. Thus, the BMP signaling is required for the M axon to terminate in the middle portion of the neuropil.

10.1523/ENEURO.0322-24.2024.f3-1 Figure 3-1 BMP signaling is active in C4da neurons. (A) Both M (left two panels) and V neurons (right two panels) express pMad (green) in the nucleus of *ppk*-tdTomato + C4da neurons (red). The nucleus can be identified by Elav staining (blue). pMad is also present in the neurites, which likely includes the pMad that are phosphorylated in the axon terminals and transported toward the nucleus. The arrowheads point at C4da somas that are identified by the presence of red puncta from *ppk*-tdTomato. Scale bar: 5  µm. (B) Expression of Tkv^ACT^ & Sax^ACT^ increases pMad levels in the C4da axon terminals. The pMad level in the GFP^+^ area was quantified as a ratio of pMad intensity to GFP intensity. Scale bar: 5  µm. Error bars: mean ± SEM. ***: p < 0.0001 (C) Expression of Tkv^ACT^ & Sax^ACT^ increases nuclear pMad levels. The pMad level in the whole soma was calculated as the ratio of pMad intensity to GFP intensity. Error bars: mean ± SEM. Scale bar: 5  µm. **: p < 0.01. Download Figure 3-1, TIF file.

10.1523/ENEURO.0322-24.2024.f3-2 Figure 3-2 Hyperactive BMP signaling causes overgrowth of C4da axon terminals. MARCM was used to express Tkv^ACT^&Sax^ACT^ in single C4da neurons. Arrowheads denote the ectopic axon branches. Single C4da neurons expressed GFP (green) while all C4da neurons expressed tdTomato (red). Scale bar: 5  µm. Download Figure 3-2, TIF file.

We then asked whether C4da neurons require Type 2 BMP receptors, Wit and Punt (Put), which mediate Dpp-to-Mad signaling (Ruberte et al., 1995; Huang et al., 2011; Follansbee et al., 2017; Gjelsvik et al., 2018; Neal et al., 2019). We first eliminated *wit* function in single C4da neurons through MARCM with the *wit^A12^* null allele. Similar to the *Mad* disruption, *wit* disruption in single M neurons shifted the axons ventrally to the position where the V axons are normally located (Fig. 3*B*; M of control vs M of *wit^A12^*, *p *= 0.0286). Consequently, the topographic difference between the M and V axon terminals was eliminated (*F*_(2,56)_ = 46.78; *p *< 0.0001; one-way ANOVA; D vs M, *p *< 0.0001; M vs V, *p *= 0.6189; Fisher's LSD). Loss of *wit* had no effect on the topographic projections of D or V neurons. This result agrees with the idea that *wit* functions upstream of *Mad* in regulating the topography. We further found that deleting *Put* in single C4da neurons through MARCM with the null allele *put^P^* (also known as *put^10460^*; Tian and Jiang, 2014) eliminated the M–V topographic difference without affecting the topographic separation of D and M neurons (Fig. 3*B*; *F*_(2,45)_ = 6.391; *p *= 0.0036; one-way ANOVA; D vs M, *p *= 0.0051; M vs V, *p *= 0.6647; Fisher's LSD). Taken together, our results demonstrate an essential contribution of BMP components for the topographically correct alignment of C4da presynaptic terminals.

### BMP signaling plays an instructive role in establishing the c4da topography

One possible model of BMP-mediated topographic mapping is that the level of BMP signaling instructs the D–V position of the C4da terminals with higher BMP signaling placing the axon more dorsally. To directly test this, we elevated the level of BMP signaling in single C4da neurons through the expression of constitutively active forms of BMP receptors. It has been reported that expression of constitutively active Type 1 BMP receptors Tkv^ACT^ and Sax^ACT^ robustly elevates BMP signaling levels (Berke et al., 2013). Indeed, expression of Tkv^ACT^ and Sax^ACT^ enhanced pMad levels in both the axon terminals and nuclei of C4da neurons (Extended Data Fig. 3-1*B,C*; axon terminals, *t *= 6.637; *p *< 0.0001; nuclei, *t *= 4.358; *p *= 0.0014; *t* test). Expression of Tkv^ACT^ and Sax^ACT^ in single C4da neurons resulted in overgrowth of their axon terminals along the A–P axis (Extended Data Fig. 3-2), as expected from a previous report (Honjo and Tracey, 2018). We found that elevating BMP signaling by expressing Tkv^ACT^ and Sax^ACT^ in single M and V neurons shifted their axon terminals dorsally, supporting the idea that the level of BMP signaling instructs the D–V position (Fig. 3*C*; *F*_(5,96)_ = 40.78; M of control vs M of OE Tkv&Sax, *p *= 0.0010; V of control vs V of OE Tkv&Sax, *p *= 0.0078).

We also examined the effect of overexpressing Wit or Put in single C4da neurons with MARCM. While overexpression of Wit slightly moved the V terminals dorsally without eliminating the M–V topographic difference (Fig. 3*D*; *p* = 0.0032), overexpression of Put eliminated the M–V topographical difference, likely by shifting the V terminals dorsally (Fig. 3*E*; Put, *F*_(2,31)_ = 20.58; *p *< 0.0001; one-way ANOVA; D vs M, *p *< 0.0001; M vs V, *p *= 0.9277; Fisher's LSD; Wit, *F*_(2,39)_ = 19.26; *p *< 0.0001; one-way ANOVA; D vs M, *p *= 0.0003; M vs V, *p *= 0.0250; Fisher's LSD). The subtle effects of Wit or Put overexpression on fine-scale topography may be due to a limitation in Dpp supply. Nevertheless, the elimination of the M–V difference by Put overexpression is consistent with the notion that BMP signaling plays an instructive role.

### C4da topography does not require nuclear pMad

**Figure 4.**
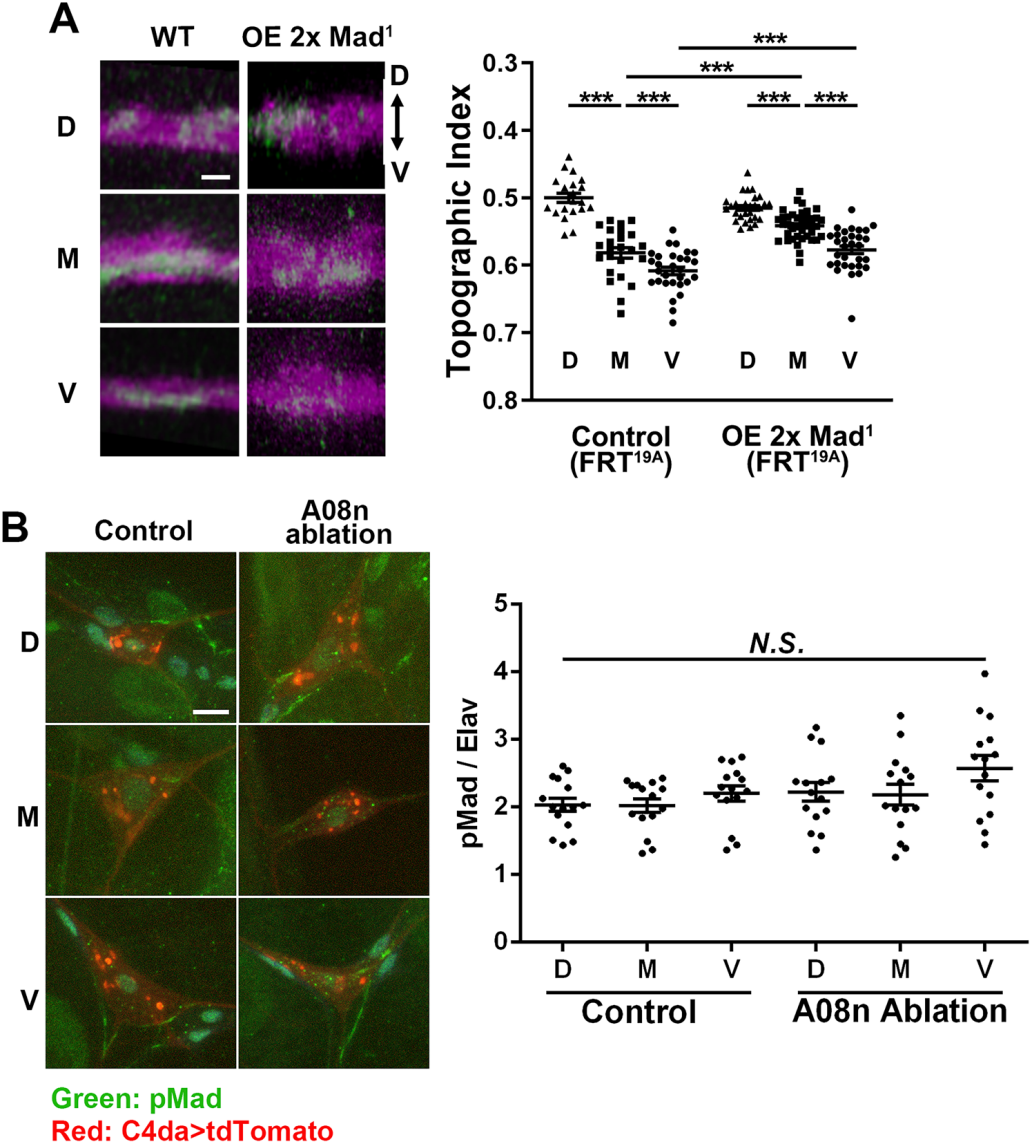
Retrograde BMP signaling does not require nuclear pMad. ***A***, Overexpression of dominant-negative Mad, Mad^1^, recapitulates the effect of BMP signaling elevation. Two copies of Mad^1^ (UAS-Mad^1^ x2) were expressed in single C4da neurons by MARCM. Left, Representative images of the dorsal–ventral view of single C4da terminals (green) in the C4da neuropil (magenta). Error bars, mean ± SEM. ***p < 0.0001. Scale bar, 2 µm. The control (FRT^19A^) is the same dataset used in Figure 1*D*. ***B***, pMad expression in C4da nuclei does not require A08n neurons. The genetic ablation of A08n neurons by Dti did not change pMad levels in C4da nuclei. The level of pMad (green) is normalized to the pan-neuronal nuclear protein Elav. C4da neurons are marked by tdTomato (red). The nuclei of C4da neurons are defined by Elav signals (not shown in the micrographs; see Extended Data Figure 3-1). One-way ANOVA was used for statistical analysis; *F* = 0.9672; *p* = 0.3885. Scale bar, 5 µm.

At the NMJ, retrograde BMP signaling controls synaptic development through pMad in the nucleus as well as that in the presynaptic terminals of motor neurons (Marques and Zhang, 2006; Sulkowski et al., 2014). In order to determine if nuclear pMad is required, we specifically disrupted nuclear pMad in single C4da neurons by expressing a dominant-negative form of pMad (Mad^1^) which lacks the DNA-binding domain while leaving the function of presynaptic pMad intact (Berke et al., 2013). This approach has been used to block NMJ growth mediated by nuclear pMad. We found that Mad^1^ expression did not ventralize the M terminals or eliminate the M–V difference (Fig. 4*A*; *F*_(2,96)_ = 48.85; *p *< 0.0001; one-way ANOVA; D vs M, *p *< 0.0001; M vs V, *p *< 0.0001; Fisher's LSD), in contrast to what was seen in *Mad^1-2^* null mutant neurons (Fig. 3*A*). Rather, Mad^1^ expression moved the terminals of both M and V neurons dorsally (M of control vs M of OE Mad^1^, *p *= 0.0002; V of control vs V of OE Mad^1^, *p *< 0.0001), similar to the effect of Tkv^ACT^ and Sax^ACT^ expression (Fig. 3*C*). This result suggests that nuclear pMad is dispensable for fine-scale C4da topography, and presynaptic Mad elevation by Mad^1^ expression is sufficient to dorsalize the axon terminals. The dispensable role of nuclear pMad is further supported by our observation that genetic ablation of A08n neurons does not change the level of nuclear pMad (Fig. 4*B*; *F*_(2,42)_ = 0.9672; *p *= 0.3885; one-way ANOVA), despite the requirement of Dpp in A08n neurons for C4da topography (Fig. 2*B*). Taken together, these results suggest that the C4da topography is not regulated by nuclear pMad, but likely by presynaptic pMad.

### Postsynaptically originated BMP signaling is required by C4da presynaptic terminals to form proper synaptic connections with postsynaptic targets

**Figure 5.**
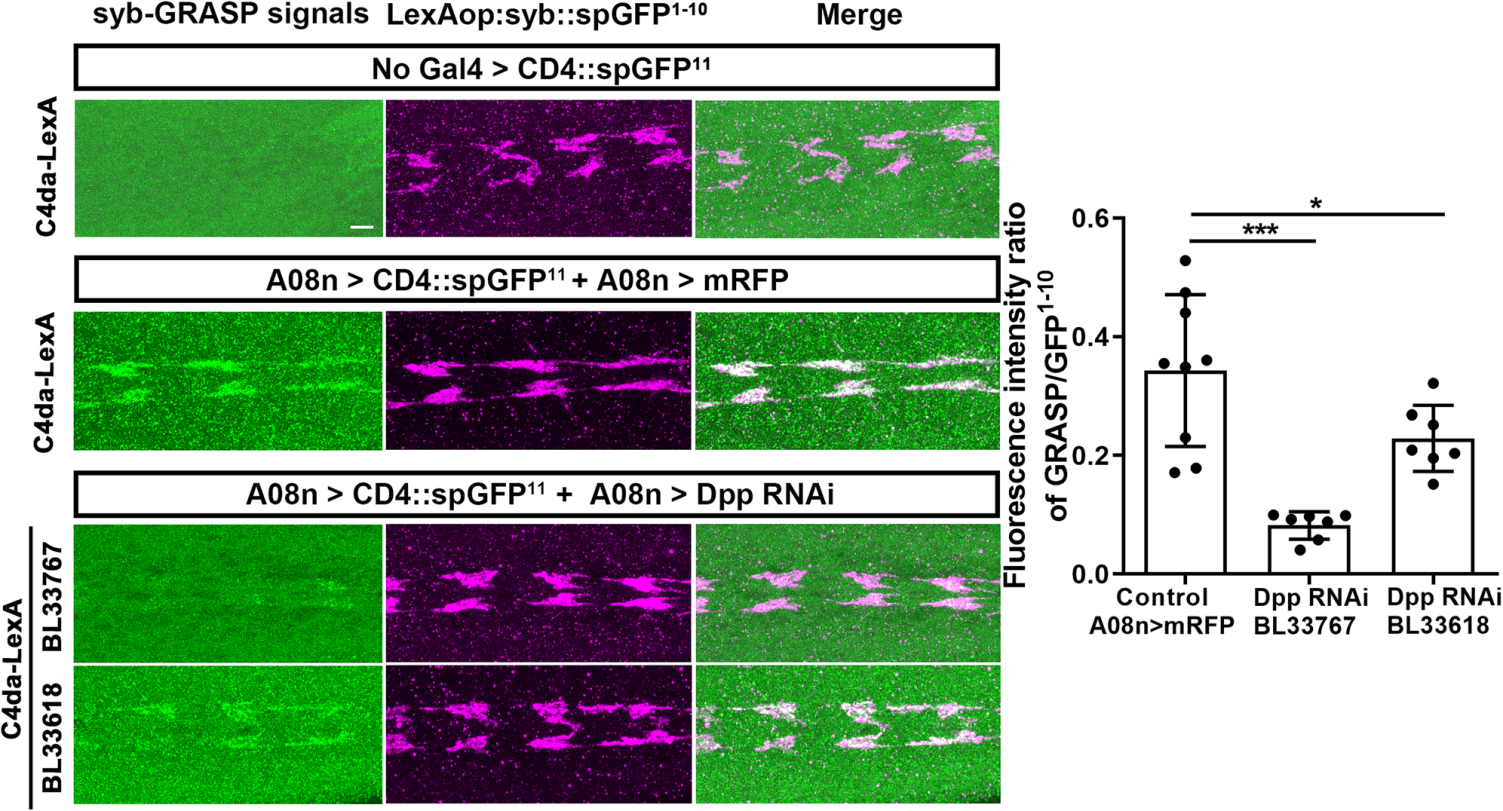
Knockdown of Dpp in A08n neurons reduces C4da→A08n synaptic contacts. Syb-GRASP was used to label C4da→A08n synaptic contacts. Syb-spGFP^1-10^ (spGFP^1-10^ fused with synaptobrevin) was expressed in C4da neurons, and spGFP^11^ was expressed in A08n neurons. The level for the GRASP signal (green) in the C4da neuropil is normalized by the level of syb-spGFP^1-10^ expression (red). The GRASP signal was stained by Alexa Flour 488, while syb-spGFP^1-10^ was stained by a secondary antibody conjugated Alexa Flour 647 to minimize bleed-through across imaging channels. UAS-mRFP was used as the control. Two independent shRNAs against Dpp were expressed in A08n neurons. One-way ANOVA was used for multigroup analysis with post hoc Fisher's LSD test. Error bars, mean ± SEM. ***p < 0.0001; *p < 0.05. Scale bar, 5 µm.

**Figure 6.**
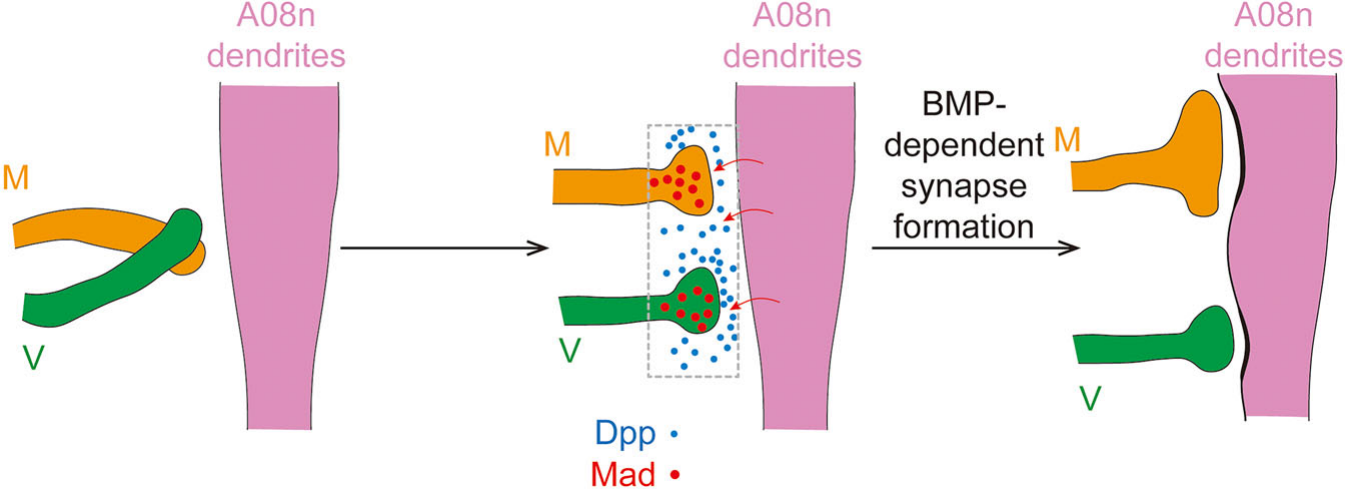
A08n-derived BMP signaling in establishing C4da topography. The axon terminals of M and V C4da neurons are initially intermingled at the first instar larvae (Yang et al., 2014). We propose a model in which local Dpp signaling from A08n neurons stabilizes M-A08n synapses more than V-A08n synapses after the first instar stage. This difference between M and V neurons might be due to potential difference in their expression of Type 2 BMP receptors (Wit and Put). We suspect that the biased stabilization of M-A08n connections recruits the M axons toward the dorsally located A08n dendrites. When a single M neuron loses BMP signaling and consequently forms less synapses with A08n neurons, the number of V-A08n synapses may surpass that of M-A08n synapses, thus impairing the dorsal positioning of the M axon.

Our results so far suggest that postsynaptically originated BMP signaling mediated by Dpp, Type 2 BMP receptors, and presynaptic pMad regulates the C4da topographic projection. Since the major function of the retrograde BMP signaling at the NMJ is to stabilize synaptic connections, we tested whether the A08n-to-C4da BMP signaling regulates topography by facilitating synapse formation. In order to examine synaptic connections of C4da neurons with A08n neurons, we used the sybGRASP technique (Macpherson et al., 2015). We expressed two complementary split GFPs in C4da (spGFP^1-10^ fused to synaptobrevin) and A08n (spGFP^11^). Wild-type larvae exhibited GRASP signals in the axon terminals where spGFP^1-10^ was expressed, which is an indication of the C4da→A08n synaptic contacts. We found that the level of GRASP signals between C4da axons and A08n dendrites decreased when Dpp was knocked down in A08n neurons by Dpp RNAi (Fig. 5; *F*_(2,20)_ = 17.61; *p *< 0.0001; one-way ANOVA; control vs BL33767, *p *< 0.0001; control vs BL33618, *p *= 0.0173; Fisher's LSD). This result demonstrates that Dpp in A08n is required for C4da axons to form proper synaptic connections with A08n neurons. Given that the Dpp RNAi also disrupted the M–V separation (Fig. 2*B*), the formation of C4da→A08n synapses may be the key determinant of fine-scale C4da topography (Fig. 6).

## Discussion

In this study, we identified BMP signaling as a mediator of transsynaptic interactions required for the topographic mapping of two adjacent sensory axons. This transsynaptic BMP signaling involves the BMP ligand Dpp from the postsynaptic neurons, as well as the BMP receptors Put, Wit, and their effector Mad in the presynaptic neurons. C4da topography not only requires all these components but also is instructed by the level of BMP signaling. We further found that blocking the postsynaptically originated BMP signaling reduces the C4da→A08n synaptic contacts. Our results favor a model in which BMP signaling enables synaptic stabilization between topographically appropriate pre- and postsynaptic terminals.

### Transsynaptic interactions in topographic projections

We found that topographic projections of fly larval nociceptors require A08n neurons, a group of postsynaptic targets of nociceptors. Contributions of postsynaptic neurons to topographic mapping have previously been observed in sensory circuits. In the fly visual system, topographic projections of photoreceptors require N-cadherin-mediated attractive interactions of the photoreceptors with their postsynaptic neurons in the lamina (C. H. Lee et al., 2001). In vertebrate visual systems, a necessary step for establishing the RGC topography is the activation (i.e., depolarization) of postsynaptic neurons by presynaptic RGCs (Cline, 1991; Kaneko and Ye, 2015). Synchronized neural activity in pre- and postsynaptic terminals leads to the activation of NMDA-type glutamate receptors in the postsynapses, which in turn stabilizes the synaptic connections and establishes fine-scale topography through steps that are yet to be defined.

Similar to the vertebrate visual system, the fine-scale topography of *Drosophila* nociceptors is instructed by neural activity of these nociceptors (Yang et al., 2014). Blocking neural activity in M neurons ventralizes their axon terminals, whereas enhancing the activity in V neurons dorsalizes their terminals. However, this role of neural activity is unlikely mediated by synchronized activation in pre- and postsynaptic partners, because the C4da topography forms even in the absence of evoked neurotransmitter release from the nociceptors (Fig. 1*D*). In contrast, our data suggest that fine-scale topography is partly regulated by spontaneous neurotransmission (Fig. 1*E*). The requirement of spontaneous neurotransmitter release in V neurons is interesting (Fig. 1*E*), given that blocking neuronal activity disrupts the M projection but not the V projection (Yang et al., 2014). This observation further supports that evoked neural activity and spontaneous neurotransmission act in separate pathways. Many studies have shown that spontaneous release of neurotransmitters from presynaptic neurons is essential for synapse formation (Andreae and Burrone, 2018). At the *Drosophila* NMJ, spontaneous release instructs the numbers of presynaptic boutons (Cho et al., 2015). Spontaneous releases might also regulate postsynaptic neurotransmitter receptor clustering at the *Drosophila* NMJ (Saitoe et al., 2001). Thus, our finding is consistent with the possibility that the synapse formation that is regulated by spontaneous neurotransmitter release is essential for fine-scale C4da topography.

### BMP signaling in establishing topography

BMP signaling is involved in many aspects of nervous system development. In vertebrate visual systems, although the contribution of BMP signaling to fine-scale topography has not been determined, an initial coarse topography is indirectly regulated by the mammalian homologs of Dpp, BMP2, and BMP4, which are expressed specifically in the dorsal retina. This dorsally restricted BMP expression drives the formation of D–V gradients that instruct coarse topography of RGC axons in a fashion similar to guidance cues (e.g., EphB2/EphrinB2; Peters and Cepko, 2002; Plas et al., 2008). In the fly visual system, Dpp-mediated BMP signaling indirectly regulates topographic projections of photoreceptors by specifying the cell fate of photoreceptor targets in the lamina (Yoshida et al., 2005).

Our results unveil a novel role of BMP signaling in topographic mapping by showing that the BMP signaling from postsynaptic neurons contributes to the fine-scale separation of adjacent presynaptic axons. Whereas broad gradients of signaling molecules across tissues are unlikely to be sufficient to differentiate two neighboring neurons, local intercellular communication at the synapse level is well suited for the regulation at the single-neuron level. In fact, a study on the fly NMJ has identified independent regulation of neighboring presynaptic terminals by retrograde transsynaptic BMP signaling (Berke et al., 2019). Our identification of the contribution of BMP signaling to local intercellular communication in cholinergic CNS synapses highlights its role as a major transsynaptic signal in both the PNS (i.e., NMJ) and the CNS, despite the differences in neurotransmitter types (glutamatergic vs cholinergic). While the muscle-to-motoneuron communication at the NMJ involves Gbb (Marques and Zhang, 2006; Berke et al., 2019), the A08n-to-C4da communication requires Dpp. This difference in BMP types may reflect that Gbb is expressed broadly in the fly VNC (Fig. 2*A*) and is possibly used for global, tissue-level regulations in the CNS. In contrast, Dpp is expressed in a small group of sparsely distributed neurons (Fig. 2*A*) and might be dedicated for fine-scale regulations at the synaptic level inside the CNS. In this regard, locally active BMP signaling through presynaptic pMad probably facilitates specific axon–target communication, in spite of the concurrent presence of different BMP ligands released from other cells, as is also the case at the NMJ (Berke et al., 2019).

Previous studies suggested that C4da neurons also express BMP ligands for autocrine BMP signaling (Follansbee et al., 2017; Gjelsvik et al., 2018). The autocrine signaling potentially contributes to the nuclear pMad activity in C4da neurons even in the absence of postsynaptic A08n neurons (Fig. 4*B*). Our finding that nuclear pMad is dispensable for C4da topography (Fig. 4*A*) supports that the tonic nuclear pMad contributes to other biological functions, such as C4da nociceptors’ sensitivity to noxious stimuli and maintenance of existing synapses (Follansbee et al., 2017; Gjelsvik et al., 2018; Honjo and Tracey, 2018; Furusawa et al., 2023), but not the axonal topography. It is noteworthy that although it is most likely that the Dpp released from postsynaptic neurons (A08n) directly act on the BMP receptors in the presynaptic neurons (C4da), our data cannot rule out the possibility that A08n-derived Dpp acts on other cells, which in turn releases BMP ligands to regulate the BMP signaling in C4da neurons.

### The requirement of synapse formation for fine-scale topography

How could transsynaptic BMP signaling contribute to fine-scale topography? Given the reduction of C4da→A08n synapses upon BMP inhibition, we propose a model whereby BMP signaling instructs topography by facilitating synapse formation between C4da axons and Dpp-expressing A08n neurons. This model is supported by a previous finding that A08n neurons form unequal numbers of synapses with each C4da subtype. EM reconstruction showed that the primary presynaptic partners of A08n neurons were the D neurons (Gerhard et al., 2017), suggesting that postsynaptic sites of A08n neurons are biased toward the dorsal portion of the synaptic areas of nociceptors. This EM study further showed that A08n neurons formed slightly more synapses with M neurons than V neurons. Such preferential selection of M axons by dorsally biased A08n dendrites could directly cause the M axons to position more dorsally than the V axons. Our model predicts that the level of BMP signaling determines the number of synapses with A08n neurons and thereby finely sets the D–V position of the C4da axon terminals. Consistently, our data demonstrate that elevating BMP signaling in C4da neurons dorsalizes both M and V axons.

It is still a mystery as to how BMP signaling places the M axon dorsally to the V axon. This might be attributable to (1) potential difference among C4da axons in the expression of Type 2 BMP receptors (Put and Wit) and (2) possible functional difference between the two receptors. Given that *wit* is required in M neurons but not in V neurons (Fig. 3*B*), we speculate that M neurons lack Put and respond to Dpp with Wit, whereas V neurons preferentially use Put to receive Dpp. It is possible that Wit induces higher BMP signaling than Put, giving the M axon higher BMP signaling and thereby more synapse with A08n. The differential expression of Type 2 receptors might be controlled by neural activity-dependent intracellular signaling pathways in C4da neurons (Yang et al., 2014).

Although C4da neurons have many types of postsynaptic partners (Vogelstein et al., 2014; Ohyama et al., 2015; Gerhard et al., 2017; Kaneko et al., 2017; Takagi et al., 2017; Yoshino et al., 2017; Burgos et al., 2018), A08n neurons are among a few postsynaptic interneurons that span the entire VNC and are thus particularly suited for ensuring a consistent D–V topography for all the segmentally repeated C4da neuropils. This unique aspect of A08n neurons, together with their dorsally biased positioning, may explain why A08n neurons are an indispensable regulator of C4da topography and one of a small group of neurons expressing Dpp.

While A08n neurons are essential for establishing the topographic projection of C4da axon terminals, they are unlikely to mediate any location-specific behavioral responses since the inputs of the three C4da converge onto them. It is possible that after A08n contributes to the topographic projection of C4da axon terminals, this fine-scale topography enables other second-order neurons to form synapses with M and V C4da neurons differently, leading to functional differences.

## Synthesis

Reviewing Editor: Lynda Erskine, University of Aberdeen Decisions are customarily a result of the Reviewing Editor and the peer reviewers coming together and discussing their recommendations until a consensus is reached. When revisions are invited, a fact-based synthesis statement explaining their decision and outlining what is needed to prepare a revision will be listed below. The following reviewer(s) agreed to reveal their identity: NONE. Note: If this manuscript was transferred from JNeurosci and a decision was made to accept the manuscript without peer review, a brief statement to this effect will instead be what is listed below.

Thank you for choosing to transfer your interesting work to eNeuro. I have carefully reviewed your rebuttal of the reviewers' comments and the manuscript. I am pleased to accept the manuscript for publication in eNeuro. Prior to publication, I have a couple of very minor points I would like you to consider:

1. The data presented in Figure 3 does not follow the same order of the descriptions of this work in the text (lines 341 - 395). Reordering the figure or the description in the text may make this section easier to follow.

2. Lines 351 - 354 are confusing as written: "We then asked whether C4da neurons require type-II BMP receptors, Wit and

Punt (Put), which mediate Dpp-to-Mad signaling (Ruberte et al., 1995; Follansbee et al.,2017), although significant contribution from the type-II receptor Wit has also been shown"
