# Supplementary figures and images for: Transsynaptic BMP Signaling Regulates Fine-Scale Topography between Adjacent Sensory Neurons
Source: eNeuro. 2024 Aug 23;11(8):ENEURO.0322-24.2024. doi: 10.1523/ENEURO.0322-24.2024 (PMC11360983; doi:10.1523/ENEURO.0322-24.2024)

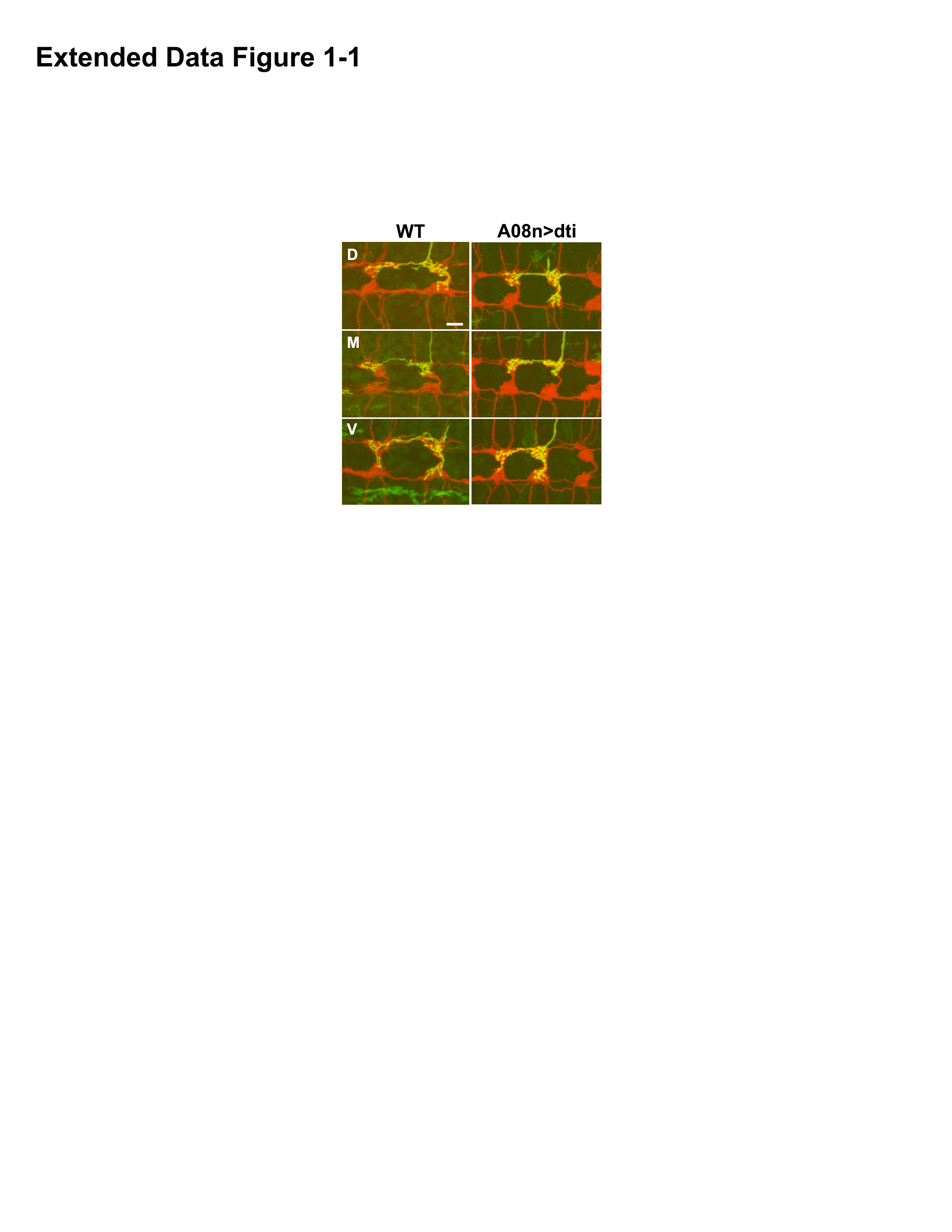

Supplement: Figure 1-1 — Genetic ablation of A08n neurons by Dti does not affect the morphology of the C4da presynaptic terminals. Single C4da neurons are labeled in green, while all C4da neurons are marked in red. Scale bars: 5 µm. Download Figure 1-1, TIF file. [file eneuro-11-ENEURO.0322-24.2024-s001.tif]

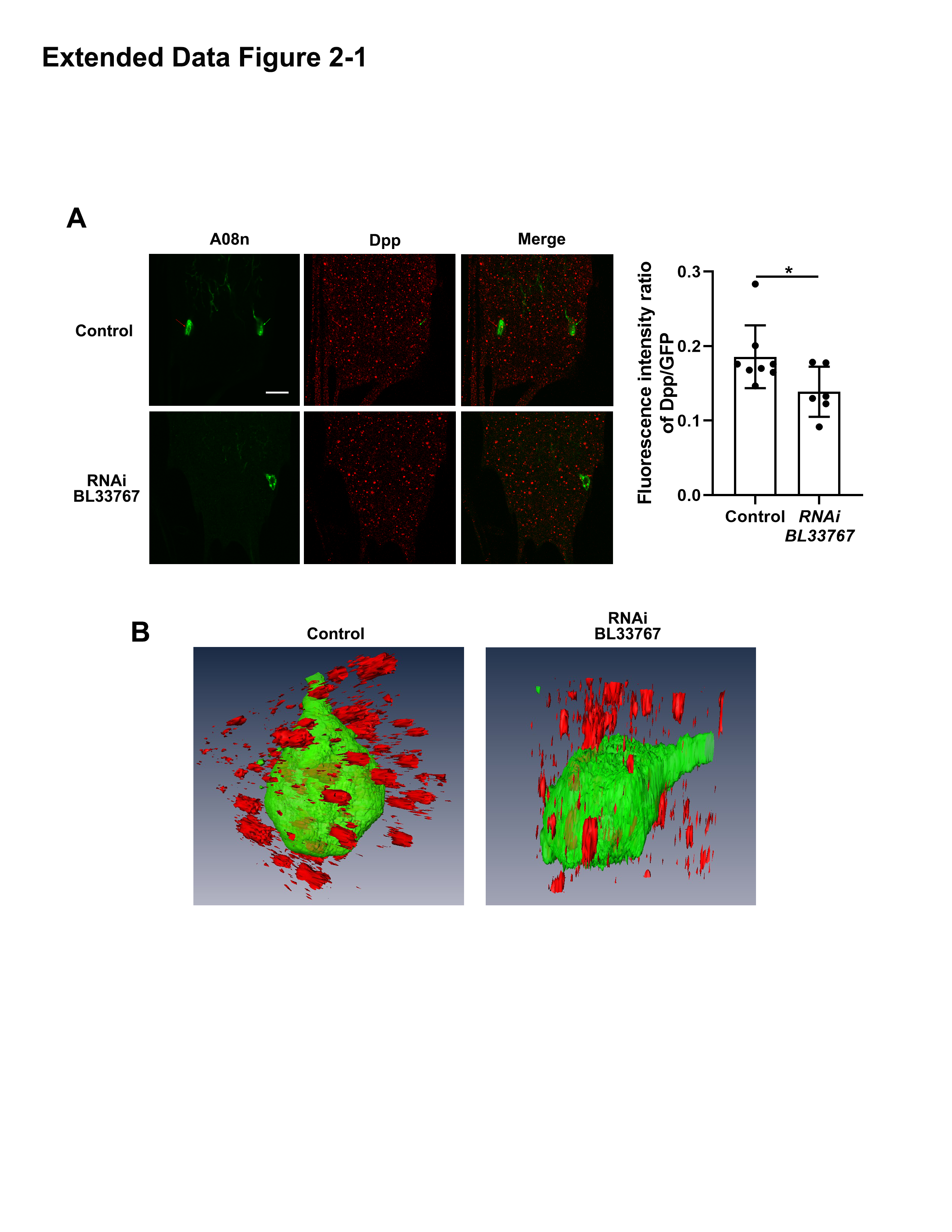

Supplement: Figure 2-1 — Dpp proteins are present in A08n soma, which decrease by RNAi knockdown. (A) Left: Dpp immunostaining of the larval VNC. Dpp is shown in red, and A08n neurons are in green. Scale bar: 10 µm. Right: quantification of Dpp levels in A08n soma. Dpp signal intensity was normalized by GFP intensity. (B) 3-D reconstruction (with Amira, FEI Visualization Sciences Group) of the immunostaining data from A. Dpp proteins (red) can be seen with in A08n soma (green). Download Figure 2-1, TIF file. [file eneuro-11-ENEURO.0322-24.2024-s002.tif]

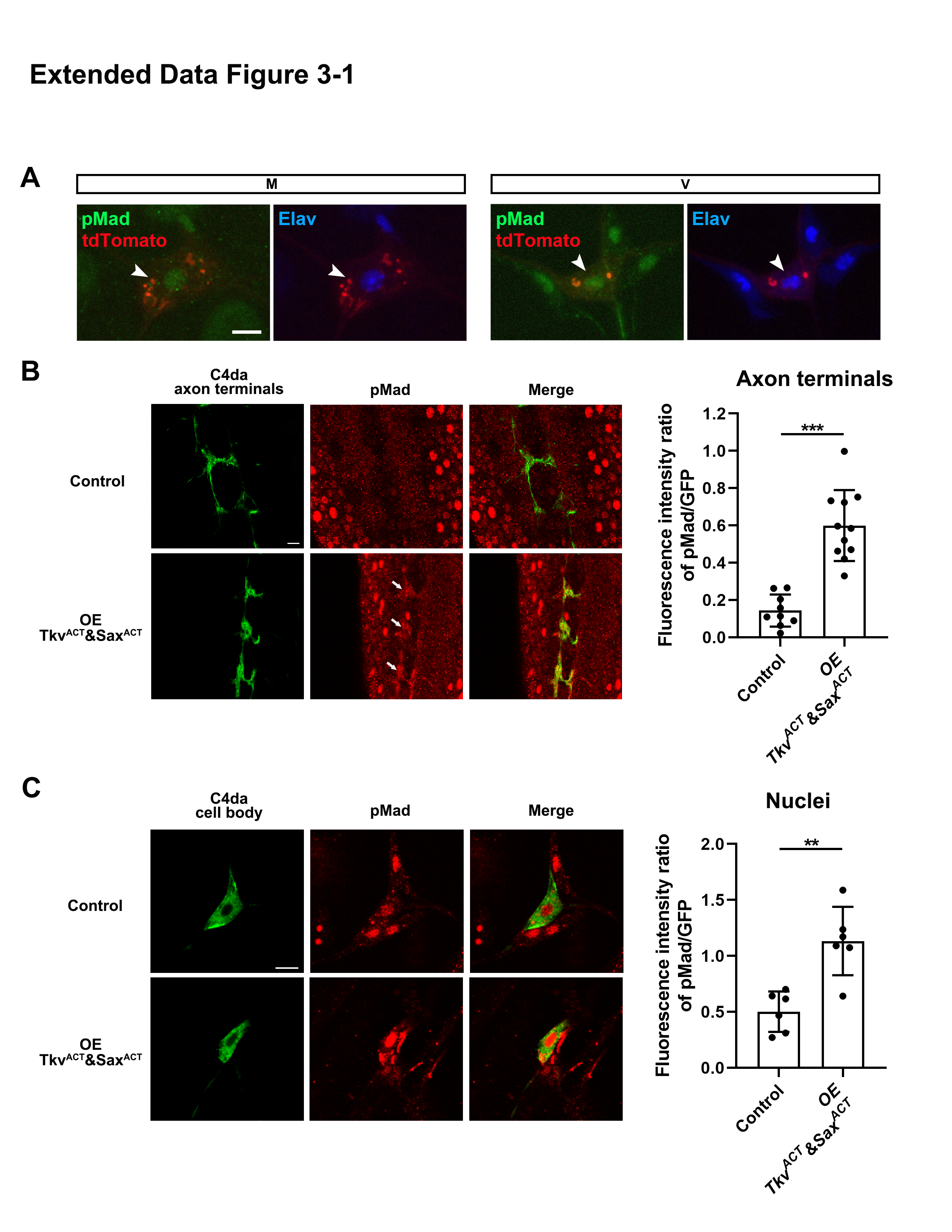

Supplement: Figure 3-1 — BMP signaling is active in C4da neurons. (A) Both M (left two panels) and V neurons (right two panels) express pMad (green) in the nucleus of ppk-tdTomato + C4da neurons (red). The nucleus can be identified by Elav staining (blue). pMad is also present in the neurites, which likely includes the pMad that are phosphorylated in the axon terminals and transported toward the nucleus. The arrowheads point at C4da somas that are identified by the presence of red puncta from ppk-tdTomato. Scale bar: 5 µm. (B) Expression of TkvACT & SaxACT increases pMad levels in the C4da axon terminals. The pMad level in the GFP+ area was quantified as a ratio of pMad intensity to GFP intensity. Scale bar: 5 µm. Error bars: mean ± SEM. ***: p < 0.0001 (C) Expression of TkvACT & SaxACT increases nuclear pMad levels. The pMad level in the whole soma was calculated as the ratio of pMad intensity to GFP intensity. Error bars: mean ± SEM. Scale bar: 5 µm. **: p < 0.01. Download Figure 3-1, TIF file. [file eneuro-11-ENEURO.0322-24.2024-s003.tif]

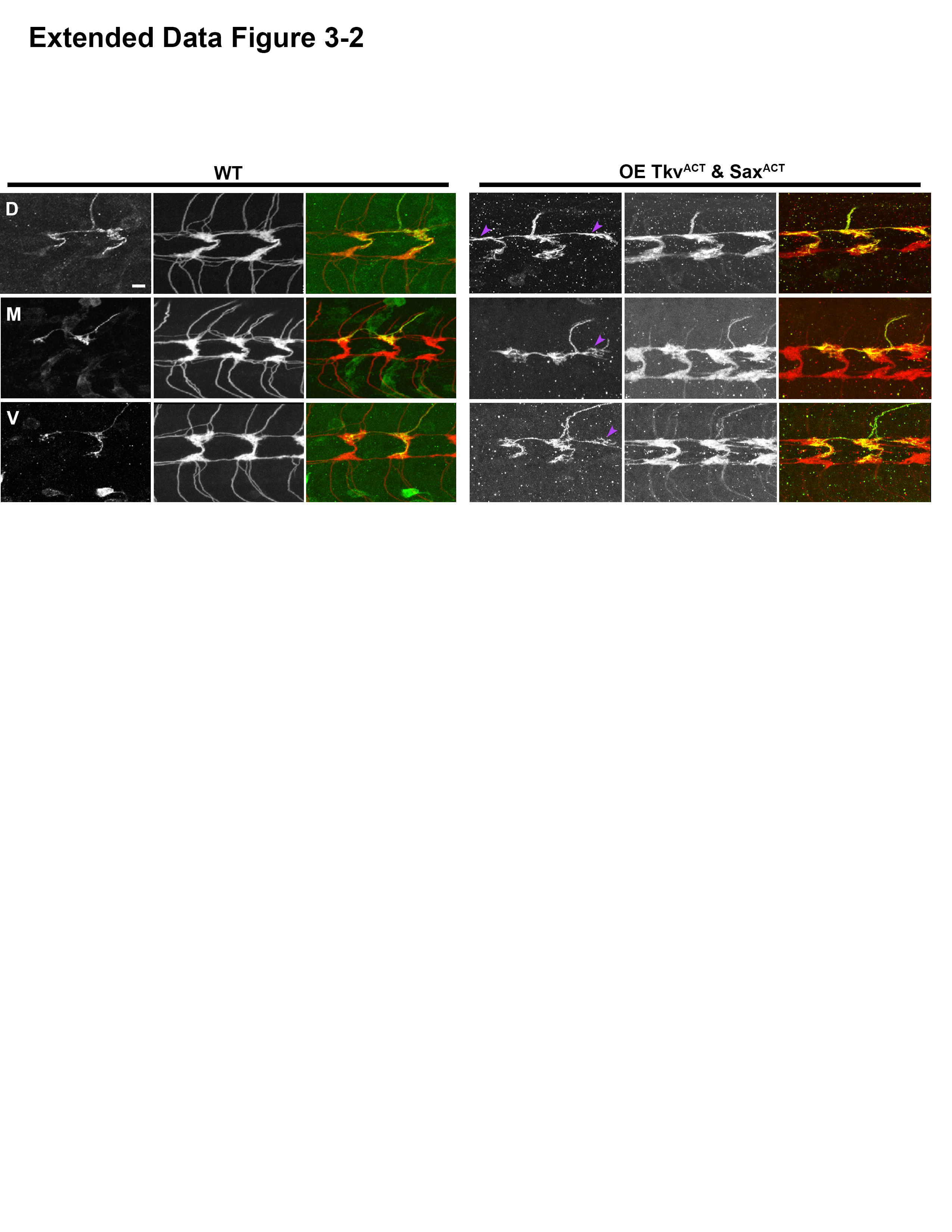

Supplement: Figure 3-2 — Hyperactive BMP signaling causes overgrowth of C4da axon terminals. MARCM was used to express TkvACT&SaxACT in single C4da neurons. Arrowheads denote the ectopic axon branches. Single C4da neurons expressed GFP (green) while all C4da neurons expressed tdTomato (red). Scale bar: 5 µm. Download Figure 3-2, TIF file. [file eneuro-11-ENEURO.0322-24.2024-s004.tif]
